# Chemicals Constituents Isolated from Cultivate *Alpinia conchigera* Griff. and Antimicrobial Activity

**DOI:** 10.21315/tlsr2020.31.1.10

**Published:** 2020-04-07

**Authors:** Mohamad Nurul Azmi Mohamad Taib, Nursyazwani Anuar, Khayriyyah Mohd Hanafiah, Aeman Ali Kudayr Al-Shammary, Mardiana Saaid, Khalijah Awang

**Affiliations:** 1 School of Chemical Sciences, Universiti Sains Malaysia, 11800 USM Pulau Pinang, Malaysia; 2 School of Biological Sciences, Universiti Sains Malaysia, 11800 USM Pulau Pinang, Malaysia; 3 Department of Chemistry, Faculty of Science, University of Malaya, 50603 Kuala Lumpur, Malaysia

**Keywords:** *Alpinia conchigera* Griff., Zingiberaceae, Antimicrobial, Methicilin Resistant *Staphlococcus aureus*, MRSA, *Alpinia conchigera* Griff., Zingiberaceae, Antimikrob, Methicilin Resistant *Staphlococcus aureus*, MRSA

## Abstract

*Alpinia conchigera* Griff. is a plant species from the family Zingiberaceae. Coloquially known as wild ginger, *Alpinia conchigera* Griff. is used as food condiment and for traditional treatment of skin diseases. Isolation studies to identify bioactive compounds of rhizomes of *Alpinia conchigera* yielded seven compounds; 1′*S*-1′-acetoxychavicol acetate (**1**), *trans*-*p*-coumaryl diacetate (**2**), *p*-hydroxycinnamyl acetate (**3**), 1′*S*-1′-hydroxychavicol acetate (**4**) *p*-hydroxybenzaldehyde (**5**), stigmasterol (**6**) and *β*-sitosterol (**7**). Compounds **1**, **2** and **5** were evaluated for antimicrobial activity against methicillin-resistant *Staphylococcus aureus* (MRSA). Among the compounds tested, Compound 1 showed good antimicrobial activity against the strain of MRSA with minimum inhibition concentration (MIC) value of 0.5 mg/mL. Meanwhile, Compounds **2** and **5** exhibited moderate activity with MIC value between 1.0 and 2.0 mg/mL. These findings indicate antimicrobial potential of 1′*S*-1′-acetoxychavicol acetate (**1**), compound derived from rhizome of *Alpinia conchigera* Griff. against MRSA, which warrant further investigation.

## Abstract (ms)

*Alpinia conchigera* Griff. merupakan spesies tumbuhan daripada famili Zingiberaceae. Lebih dikenali sebagai lengkuas liar, *Alpinia conchigera* Griff. sering digunakan sebagai perasa makanan dan rawatan tradisional penyakit kulit. Pemencilan ke atas rizom *Alpinia conchigera* telah menghasilkan tujuh sebatian; 1′*S*-1′-asetoksikhavikol asetat (**1**), *trans*-*p*-koumaril diasetat (**2**), *p*-hidroksisinamil asetat (**3**), 1′*S*-1′-hidrosikhavikol asetat (**4**), *p*-hidroksibenzaldehid (**5**), stigmasterol (**6**) dan *β*-sitosterol (**7**). Sebatian **1**, **2** dan **5** telah dinilai untuk aktiviti antimikrobial terhadap *Staphylococcus aureus* iaitu MRSA. Antara sebatian yang dinilai, sebatian **1** menunjukkan aktiviti antimikrobial yang kuat terhadap MRSA dengan nilai MIC 0.5 mg/mL. Sementara itu, Sebatian **2** dan **5**, masing-masing menunjukkan aktiviti antimikrobial sederhana dengan nilai MIC di antara 1.0 dan 2.0 mg/mL. Dapatan ini menunjukkan potensi antimikrob sebatian 1′*S*-1′-asetoksikhavikol asetat (**1**) yang berasal dari rizom *Alpinia conchigera* Griff. terhadap MRSA yang harus dikaji selidik dengan lebih lanjut.

**Highlights**

- Phenylpropanoids were successful isolate as the major components in *Alpinia conchigera*.
- 1′*S*-1′-acetoxychavicol acetate exhibited good antimicrobial activity can be further evaluated as a potential candidate for treatment against MRSA.
- The results provide some evidence to support the traditional application of this herb in treatment of skin infection as reported in Kelantan, East Coast Malaysia.

## INTRODUCTION

**Figure 1:**
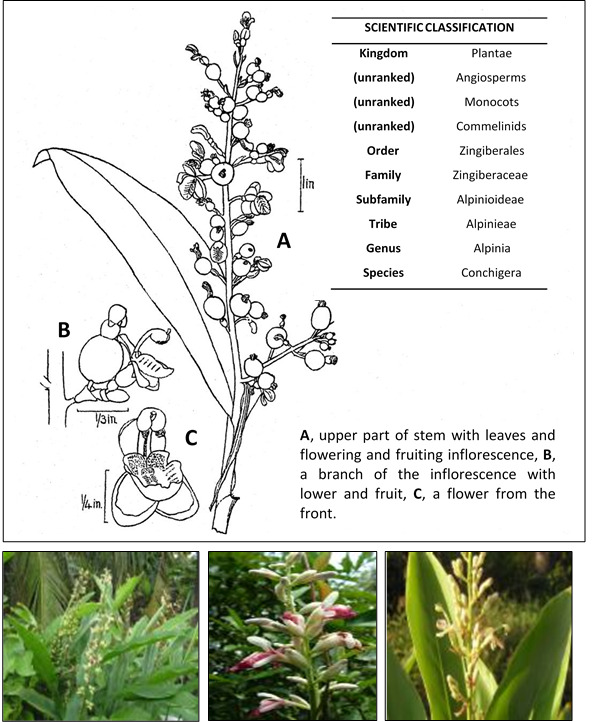
*Alpinia conchigera* Griff.

*Alpinia conchigera* (Fig. 1) is a Malaysian wild-ginger and also known locally as “lengkuas ranting”, “lengkuas kecil”, “lengkuas padang”, “lengkuas genting” or “chengkenam” (Ibrahim *et al.* 2008; Henderson 1954; Ridley 1909). It is an herbaceous perennial 2 to 5 feet tall and usually found in eastern Bengal and southwards to Peninsular Malaysia and Sumatera (Burkhill 1966; Smith 1990). Traditionally, all parts of plants such as rhizomes, seeds, flowers and leaves are used medicinally and claimed to have a range effect to treat many physical illnesses. In some states of Peninsular Malaysia, the rhizome of *Alpinia conchigera* are consumed to treat skin disease (Ibrahim *et al.* 2009) and also used as food condiment (Victorio 2011). Besides that, it is also advocated for the treatment of diabetes in Thailand (Wongsatit & Ampol 2003).

There are few studies reporting on the chemical constituents from *Alpinia conchigera* Griff. Aziz *et al*. (2013) reported seven known phenylpropanoids isolated from *Alpinia conchigera*; chavicol acetate, *p*-hydroxycinnamaldehyde, 1′*S*-1′-acetoxychavicol acetate, *trans-p*-coumaryl diacetate, 1′*S*-1′-acetoxyeugenol acetate, 1′-hydroxychavicol acetate and *p*-hydroxycinnamyl acetate. Of these, 1′*S*-1′-acetoxychavicol acetate was found as the major constituent and has been reported to bear a variety of important biological activities such as antitumor, anti-inflammatory, antifungal, antioxidative, anti-human immunodeficiency virus (HIV) and xanthine oxidase inhibitory activity (Yasuhara *et al.* 2009). Besides that, the presence of 1′*S*-1′-acetoxychavicol acetate and 1′*S*-1′-acetoxyeugenol acetate in the rhizome were found to induce apoptosis in oral squamous carcinoma cells (HSC-4) and human breast cancer cells (MCF-7) (Awang *et al.* 2010; Hasima *et al.* 2010).

For the past few decades, antimicrobial resistance has become a serious threat to global public health as the number of infectious diseases by antibiotic resistance pathogens increased significantly. Methicillin-resistant *Staphylococcus aureus* (MRSA) is the drug resistant form of *Staphylococcus aureus*, which are normal skin flora that can opportunistically infect skin and mucous tissues. Traditionally known for its role in nosocomial infections, MRSA is major cause of morbidity and mortality in hospital worldwide (Lakhundi & Zhang 2018). However, increasing prevalence of community-acquired MRSA has also been reported in recent years. As MRSA is resistant to the first-line beta-lactam antibiotics, including methicillin, oxacilin, penicillin and amoxycilin (Baba *et al.* 2016), patients often need to be treated with last-resort drugs such as vancomycin, which possess higher toxicity. Hence, there is an urgent need to explore new antimicrobial compounds that may have lower toxicity such as naturally derived compounds. In the work reported herein, we investigate the chemical constituents isolated from cultivated *Alpinia conchigera* Griff. and their antimicrobial activity against MRSA.

## MATERIAL AND METHODS

### General

NMR spectra were obtained using Bruker Advance 500 (500 MHz for ^1^H NMR, 125 MHz for ^13^C NMR) spectrometer system. Data were analysed via Top Spin software package. Spectra were referenced to TMS or residual solvent (CDCl_3_ = 7.26 ppm in ^1^H NMR and 77.0 ppm in ^13^C NMR). ^1^H NMR spectroscopic data is reported as follows: chemical shift (relative integral, multiplicity [s – singlet, d – doublet, dd–doublet of doublets, spin – spin coupling constant (*J* in Hertz, Hz), identifier. Mass spectra were carried out on Perkin Elmer GC-MS spectrometer (Perkin Elmer Clarus 600T GCMS System). Fourier transform infrared spectra were obtained using a Perkin-Elmer Spectrum 1000 series Fourier Transform IR spectrometer with Universal Attenuated Total Reflectance (Diamond) sampling accessory. Absorption maxima are expressed as wave numbers (cm^−1^).

### Material

The rhizome of *Alpinia conchigera* Griff. was collected in Jeli, Kelantan and cultivate in Ulu Langat, province of Selangor, west-coast of Peninsular Malaysia and the series number was given as KL5049 (Fig. 1). This species was identified by Professor Dr. Halijah Ibrahim from Institute of Biological Sciences, University of Malaya and a voucher specimen were identified and deposited in the herbarium of the Department of Chemistry, University of Malaya, Kuala Lumpur.

### Gas Chromatography (GC)

GC analysis was performed on Agilent Technologies 7890A GC System, fitted with 30 m × 0.32 mm i.d × 0.25 μm HP-5 capillary column, using nitrogen as the carrier gas. The oven temperature was programmed from 60°C to 280°C at 20°C/min and the end temperature was held for 1 min.

### Gas Chromatography Mass Spectrometry (GCMS)

Analyses were carried out on Perkin Elmer Clarus 600T mass spectrometer, directly coupled to Perkin Elmer Clarus 600 gas chromatograph with 30 m × 0.25 mm i.d × 0.25 μm HP-5MS capillary column, using helium as the carrier gas. The oven temperature was programmed from 60°C to 280°C at 20°C/min and the end temperature was held for 1 min.

### Extraction and Isolation

The dry and ground rhizomes (1.5 kg) were extracted using maceration method with *n*-hexane, dichloromethane (DCM) and methanol (MeOH) successively. The extracts were dried *in vacuo* using rotary evaporator. The *n*-hexane and DCM were further subjected to chemical isolation and antibacterial studies. These extracts were subjected to column chromatography (CC) on silica gel using stepwise gradient system (*n*-hexane to ethyl acetate).

The crude extracts of *n*-hexane (1.95 g) was fractionated using silica gel CC. The extract was dry packing on celite and loaded on a 100 × 15 cm glass column packed with 50 g of silica gel. The column was eluted using a *n*-hexane: ethyl acetate gradient (1:0 to 5:5 v/v) to give 71 fractions. The eluate was collected in the test tube and combined based on the basic of TLC profile. Combination of eluent from fraction 1 (FrA1) gave compound **1** (0.5 g, yellowish oil), compound **5** (1.3 mg, yellowish oil) and compound **3** (9.0 mg, yellowish oil). Fraction 3 (FrA1.3) was re-chromatographed using 60 × 8 cm glass column packed with 5 g silica gel and eluted with *n*-hexane: ethyl acetate (1:0 to 6:4 v/v). From this process 68 fractions were obtained. Combination of eluent from fraction FrA1.3.2 gave compound **2** (0.2 g, yellowish oil). Meanwhile, the crude extracts of DCM (9.60 g) was subjected to CC. The eluate was collected and combined into four fractions (FrB1 to FrB4). Re-column fractions FrB2 gave compound **1** (2.2 g, yellowish oil) and compound **2** (0.2 g, yellowish oil). While analysis of fraction FrB3 gave mixture of compounds **6** and **7** (0.1 g, white solid) and fraction FrB4 gave a compound **4** (1.0 mg, yellow oil), respectively.

All compounds were successfully characterised using spectroscopic methods and by comparing the data analysis with the literature values. The list of chemical compounds isolated from *Alpinia conchigera* Griff. was shown in Fig. 3 and Tables 2–5.

1′*S*-1′-acetoxychavicol acetate (**1**):

Yellowish oil. IR (cm^−1^): 2932, 1760 (C=O ester),1607, 1370, 1234 (C-O ester), 1204, 1018. ^1^H NMR (500 MHz, CDCl_3_, δ ppm) 2.08 (3H, s, H5′), 2.27 (3H, s, H2″), 5.22 (2H, dd, *J* = 14.6, 10.5 Hz, H3′), 5.98 (1H, m, H2′), 6.23 (1H, d, *J* = 5.8 Hz, H1′), 7.03 (2H, d, *J* = 8.8 Hz, H3, H5), 7.33 (2H, d, *J* = 8.8 Hz, H2, H6). ^13^C NMR (125 MHz, CDCl_3_, δ ppm) 21.2 (C5′), 21.3 (C2″), 75.6 (C1′), 117.2 (C3′), 121.7 (C3, C5), 128.5 (C2, C6), 136.1 (C2′), 136.5 (C1), 150.5 (C4), 169.4 (C1″), 169.7 (C4′). GCMS m/z (EI) 77, 103, 132, 150, 192, 234 (M^+•^)

*trans*-*p*-coumaryl diacetate (**2**)

Yellowish oil. IR (cm^−1^): 2918, 1705 (C=O ester), 1464, 1229 (C-O ester). ^1^H NMR (500 MHz, CDCl_3_, δ ppm) 2.10 (3H, s, H2″), 2.29 (3H, s, H5′), 4.70 (2H, d, *J* = 5.1, H3′), 6.22 (1H, m, H2′), 6.60 (1H, d, *J* = 15.9 Hz, H1′), 7.03 (2H, d, *J* = 8.6 Hz, H3, H5), 7.38 (2H, d, *J* = 8.6 Hz, H2, H6). ^13^C NMR (125 MHz, CDCl_3_, δ ppm) 20.9 (C2″), 21.0 (C5′), 64.8 (C3′), 121.5 (C3, C5), 121.6 (C2′), 127.5 (C2, C6), 133.0 (C1′), 133.9 (C1), 150.3 (C4), 169.3 (C1″), 170.7 (C4′). GCMS m/z (EI) 121, 133, 149, 150, 192, 234 (M^+•^)

*p*-hydroxycinnamyl acetate (**3**)

Yellowish oil. IR (cm^−1^): 3400 (OH stretch), 1737 (C=O ester). ^1^H NMR (500 MHz, CDCl_3_, δ ppm) 2.30 (3H, s, 5′), 4.31 (2H, d, *J* = 5.6 Hz, H3′), 6.32 (1H, m, H2′), 6.58 (1H, d, *J* = 15.9 Hz, H1′), 7.03 (2H, d, *J* = 8.7 Hz, H3, H5), 7.38 (2H, d, *J* = 8.7 Hz, H2, H6). ^13^C NMR (125 MHz, CDCl_3_, δ ppm) 21.1 (C5′), 63.6 (C3′), 121.7 (C3, C5), 127.4 (C2, C6), 128.7 (C2′), 130.1 (C1′), 134.5 (C4), 150.1 (C1), 169.5 (C4′). GCMS m/z (EI) 77, 94, 107, 150, 192 (M^+•^)

1′*S*-1′-hydroxychavicol acetate (**4**)

Yellowish oil. IR (cm^−1^): 3080 (OH stretch), 1756 (C=O ester), 1201(C-O). ^1^H NMR (500 MHz, CDCl_3_, δ ppm) 2.32 (3H, s, H2″), 5.18 (2H, dd, *J* = 17.1, 9.3 Hz, H3′), 5.32 (1H, d, *J* = 6.7, H1′), 6.01 (1H, m, H2′), 7.05 (2H, d, *J* = 8.5 Hz, H3, H5), 7.36 (2H, d, *J* = 8.5 Hz, H2, H6). ^13^C NMR (125 MHz, CDCl_3_, δ ppm) 21.2 (C2″), 115.4 (C1′), 121.5 (C3, C5), 127.3 (C2, C6), 140.0 (C1), 140.1 (C2′), 150.2 (C4), 169.4 (C1″). GCMS m/z (EI) 55, 65, 77, 95, 107, 121, 133, 150, 192 (M^+•^)

*p*-hydroxybenzaldehyde (**5**)

Yellowish oil. IR (cm^−1^): 3369 (OH stretch), 1683 (C=O aldehyde), 860. ^1^H NMR (500 MHz, CDCl_3_, δ ppm) 6.97 (2H, d, *J* = 8.8 Hz, H2, H6), 7.81 (2H, d, *J* = 8.8 Hz, H3, H5), 9.82 (1H, s, H1′). ^13^C NMR (125 MHz, CDCl_3_, δ ppm) 116.1 (C2, C6), 129.7 (C1), 132.6 (C3, C5), 161.8 (C4), 191.4 (C1′). GCMS m/z (EI) 65, 74, 93, 110, 122 (M^+•^)

Stigmasterol (**6**)

White solid. IR (cm^−1^): 3430 (OH stretch), 1653 (C=C) 1463, 1049. ^1^H NMR (500 MHz, CDCl_3_, δ ppm) 0.67 (3H, s, H18), 0.78 (3H, d, *J* = 6.5 Hz, H27), 0.81 (3H, t, *J* = 7.8 Hz, H29), 0.83 (3H, d, *J* = 6.5 Hz, H26), 0.92 (1H, m, H9),1.00 (3H, s, H19), 1.01 (1H, m, H14), 1.01 (3H, s, H21), 1.06 (1H, m, H1b), 1.06 (1H, m, H15b), 1.13 (1H, m, H17), 1.15 (1H, m, H28b), 1.15 (1H, m, H12b),1.43 (1H, m, H28a), 1.46 (1H, m, H8), 1.50 (1H, m, H2b),1.50 (1H, m, H7b),1.50 (1H, m, H11), 1.52 (1H, m, H24), 1.53 (1H, m, H25), 1.56 (1H, m, H15a), 1.65 (1H, m, H16), 1.82 (1H, m, H1a), 1.82 (1H, m, H2a), 1.98 (1H, m, H7a),1.98 (1H, m, H12a), 2.01 (1H, m, H20), 2.22 (1H, m, H4b), 2.29 (1H, m, H4a), 3.50 (1H, m, H3), 5.03 (1H, dd, *J* = 15.6, 8.7 Hz, H23), 5.17 (1H, dd, *J* = 15.6, 8.7 Hz, H22), 5.33 (1H, m, H6). ^13^C NMR (125 MHz, CDCl_3_, δ ppm) 12.0 (C18), 12.2 (C29), 19.0 (C27), 19.4 (C19), 21.2 (C26), 21.1 (C11), 21.1 (C21), 25.4 (C28), 24.3 (C15), 28.9 (C16), 31.6 (C2), 31.9 (C25), 31.9 (C7), 31.9 (C8), 36.5 (C10), 37.2 (C1), 39.7 (C12), 40.5 (C20), 42.3 (C4), 42.3 (C13), 50.1 (C9), 51.2 (C24), 55.9 (C17), 56.8 (C14), 71.8 (C3), 121.7 (C6), 129.2 (C23), 138.3 (C22), 140.7 (C5). GCMS m/z (EI) 119,145, 173, 199, 271, 300, 327, 369, 412 (M^+•^)

*β*-sitosterol (**7**)

White solid. IR (cm^−1^): 3430 (OH stretch), 1463, 1049. ^1^H NMR (500 MHz, CDCl_3_, δ ppm) 0.67 (3H, s, H18), 0.81 (3H, d, *J* = 6.5 Hz), 0.83 (3H, d, *J* = 6.5 Hz), 0.85 (3H, t, *J* = 7.8 Hz). 0.92 (1H, m, H24), 0.92 (1H, m, H9), 0.92 (3H, s, H21), 1.00 (3H, s, H19), 1.01 (1H, m, H14), 1.01 (1H, m, H22b), 1.06 (1H, m, H1b), 1.06 (1H, m, H15b), 1.13 (1H, m, H17), 1.15 (1H, m, H12b), 1.15 (1H, m, H23), 1.24 (1H, m, H28), 1.30 (1H, m, H22a), 1.35 (1H, m, H20), 1.46 (1H, m, H8), 1.50 (1H, m, H2b), 1.50 (1H, m, H7b), 1.50 (1H, m, H11), 1.56 (1H, m, H15a), 1.65 (1H, m, H16), 1.65 (1H, m, H25), 1.82 (1H, m, H1a), 1.82 (1H, m, H2a), 1.98 (1H, m, H7a), 1.98 (1H, m, H12a), 3.50 (1H, m, H3), 2.22 (1H, m, H4b), 2.29 (1H, m, H4a), 5.33 (1H, m, H6). ^13^C NMR (125 MHz, CDCl_3_, δ ppm) 11.8 (C18′), 12.0 (C29′), 18.8 (C27′), 19.4 (C19′), 19.8 (C26′), 21.1 (C11′), 21.1 (C21′), 23.0 (C28′), 24.3 (C15′), 26.0 (C23′), 28.2 (C16′), 29.1 (C25′), 31.6 (C2′), 31.9 (C8′), 31.9 (C7′), 33.9 (C22′), 36.1 (C20′), 36.5 (C10′), 37.2 (C1′), 39.8 (C12′), 40.2 (C4′), 42.3 (C13′), 45.8 (C24′), 50.1 (C9′), 56.0 (C17′), 56.7 (C14′), 71.8 (C3′), 121.7 (C6′), 140.7 (C5′). GCMS m/z (EI) 55, 81, 105, 133, 159, 187, 213, 273, 382, 414 (M^+•^)

### Chemical Transformation of 1′*S*-1′-acetoxychavicol acetate (1) into *trans*-*p-*coumaryl diacetate (2)

The chemical transformation of (**1**) into (**2**) was done by modification of Azuma’s procedure (Azuma *et al.* 2006). 1′*S*-1′-acetoxychavicol acetate (**1**) (0.2 g) and sodium acetate (0.2 g) were dissolved in acetic acid (10 mL). After stirring overnight at 60°C, the mixture was neutralised with saturated aqueous NaHCO_3_ and extracted with EtOAc (3 × 10 mL). The organic layer was dried with Na_2_SO_4_ and the solvents were removed *in vacuo* using rotary evaporator. The obtained residue was analysed directly without purification and the spectroscopic data was identical to that of natural *trans*-*p*-coumaryl diacetate (**2**).

### Preparation of Bacterial Inoculum

MRSA (American Type Culture Collection 43300) was cultured in Brain Heart Infusion Broth overnight in a rotary shaker at 37°C, centrifuged at 200 rpm. The cultures pellet was then resuspended in an equal volume of phosphate buffer solution (PBS) solution and the optical density was adjusted to 0.05 at 600 nm, corresponding to approximately 1 × 10^7^ colony forming units (CFU) per mL.

### Antimicrobial Activity

Antimicrobial activity was measured by using broth microdilution technique on sterile 96-well microtiter plates. Briefly, all the tested compounds were dissolved in dimethyl sulfoxide (DMSO) to a final concentration of 31.25 μg/mL. 100 μL of double strength Muller Hilton broth was distributed to each row of the wells. Then, 100 μL of each test compound solution was serially diluted across the wells in duplicate, starting from a concentration of 4000 μg/mL to 31.25 μg/mL, the final well of each row left without addition of test compound to serve as a negative control. In addition, tetracycline was used as positives control as comparison for antimicrobial potential.

5 μL of bacterial suspension adjusted to a 0.5 MacFarland turbidity standard was inoculated into each antimicrobial-containing and control (antimicrobial free) wells. Plates were wrapped loosely with a cling film to avoid suspension dehydration and was incubated at 37°C, under aerobic conditions, for 24 h. At the end of the incubation period, 50 μL tetrazolium solution was added into all wells and the plates were re-incubated for an additional hour. The minimum inhibitory concentration (MIC) determined as the last well before the tetrazolium color changes from yellow to purple indication microbial growth.

## RESULTS AND DISCUSSION

**Figure 2:**
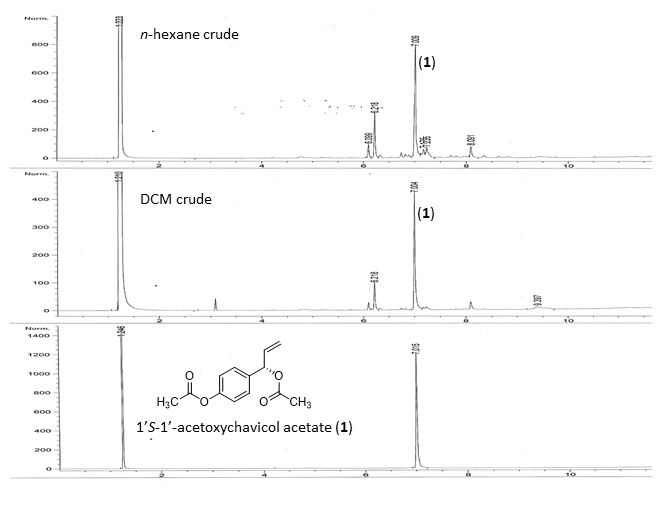
Gas chromatography chromatograms for *n*-hexane and dichloromethane extracts.

Maceration extraction of the rhizomes yielded 1.95 g (0.13% w/w) of *n*-hexane extracts, 9.60 g (0.64% w/w) of DCM extracts and 11.7 g (0.78% w/w) of MeOH extracts (Table 1). The *n*-hexane dan DCM crudes were injected into a GC system to give the chromatograms (Fig. 2), in which compound **1** gave a strong peak at *t_R_* = 7.00 min. From these chromatograms, it showed that compound **1** was the major component in *Alpinia conchigera* Griff.

**Figure 3:**
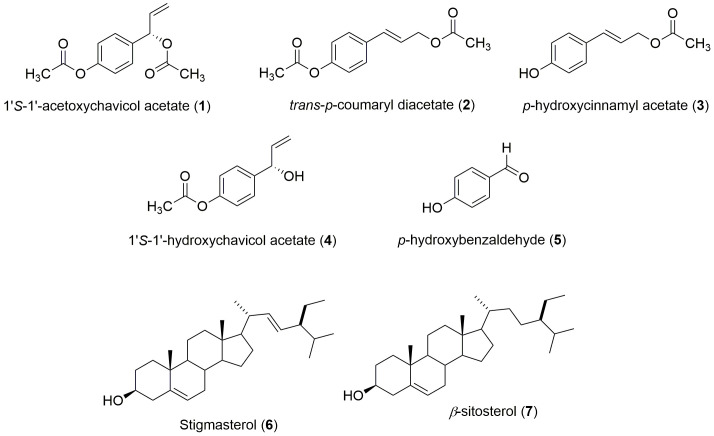
The structure of compounds **1**–**7** isolated from the rhizomes of *Alpinia conchigera* Griff.

Seven compounds were successfully isolated from the rhizomes of *Alpinia conchigera* Griff (Fig. 3 and Table 2). The isolation of *n*-hexane crude extract afforded four phenylpropanoids compounds which known as 1′*S*-1′-acetoxychavicol acetate (**1**), *trans*-*p*-coumaryl diacetate (**2**) and *p*-hydroxycinnamyl acetate (**3**), and one phenolic compound; *p*-hydroxybenzaldehyde (**5**). Meanwhile, the DCM crude extract gave three phenylpropanoids; 1′*S*-1′-acetoxychavicol acetate (**1**), *trans*-*p*-coumaryl diacetate (**2**) and 1′*S*-1′-hydroxychavicol acetate (**4**) and a mixture of triterpenes; stigmasterol (**6**) and *β*-sitosterol (**7**). The NMR spectra results are presented in Table 3–5, respectively.

**Figure 4:**
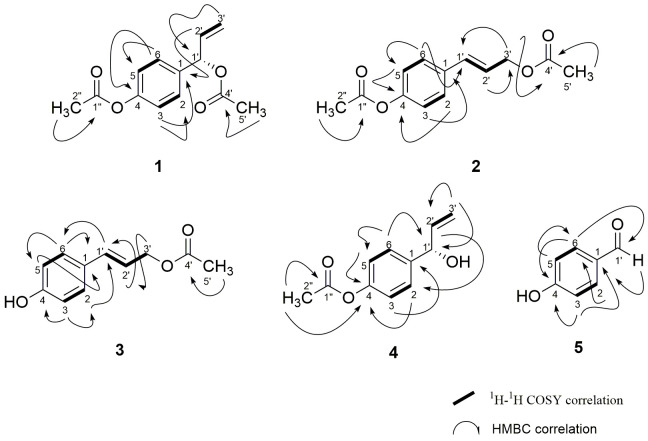
HMBC and ^1^H-^1^H COSY correlation of compounds **1**–**5**.

Compound **1** was obtained as a yellowish oil. The IR spectrum of compound **1** showed absorption bands indicative of an ester functionality at 1760 cm^−1^ (C=O ester) and 1234 cm^−1^ (C-O ester). The GC-EIMS of compound **1** displayed a molecular ion peak at m/z 234 and six important fragment ions at m/z 192 [M^+^-CH_2_=C=O], 150 [M^+^-CH_2_=C=O], 149, 132 [M^+^-C=O], 104 and 77 [M^+^-CH_2_=CH^−^]. The presence of this phenylpropanoid skeleton was disclosed by analysis of ^1^H NMR, ^13^C NMR, COSY and HMBC spectral data (Table 3 and Fig. 4). The ^1^H NMR spectrum showed two methyl at δ_H_ 2.08 (3H, *s*) and δ_H_ 2.27 (3H, *s*) corresponding to the methyl groups attached directly with the ester carbonyl groups, C-5′ and C-2″ respectively. The olefin proton H-2′ and H-3′ appeared as a multiplet and doublet of doublet at δ_H_ 5.98 (1H, *m*) and δ_H_ 5.26 (2H, *dd*, *J* = 10.5, *J*′ = 14.6). A set of doublets corresponding to four proton signals of H-2/H-6 and H-3/H-5 were appeared at δ_H_ 7.33 (2H, *d*, *J* = 8.8) and δ_H_ 7.03 (2H, *d*, *J* = 8.8) respectively indicating that the aromatic ring is 1,4-disubstituted. The COSY ^1^H-^1^H spectrum nicely displays the connectives within the allylic moiety H-1′, H-2′ and H-3′, demonstrating that in a COSY spectrum not only vicinal and germinal but also allylic spin coupling constants yield cross peaks (Fig. 4). The ^13^C NMR spectrum reveals a typical pattern of *para*-substituted aromatic compound. The assignment for two ester carbonyl carbon atoms C-1″ and C-4′ at δ_C_ 169.4 and δ_C_ 169.7 are obvious. The peak at δ_C_ 75.6 could be assigned to methane C-1′ attached to the terminal alkene. The peak at δ_C_ 136.1 might be attributed to C-2′, while at δ_C_ 117.2 to C-3′ of the terminal vinyl group. Furthermore, the peak at δ_C_ 136.5 and δ_C_ 150.5 might be attributed to the quaternary carbons, C-1 and C-4 respectively. In addition, the *sp*^2^ carbon at position C-2/C-6 and C-3/C-5 resonated at δ_C_ 128.5 and δ_C_ 121.7. Comparison of the observed data with the literature value (Watanabe *et al.* 1995) confirmed that compound **1** was 1′*S*-1′-acetoxychavicol acetate.

Compound **2** was obtained as yellowish oil. The IR spectrum of compound **2** showed absorption bands indicative of an ester functionality at 1705 cm^−1^ (C=O ester) and 1229 cm^−1^ (C-O ester). The molecular formula of C_13_H_14_O_4_ was determined by GC-EIMS at m/z 234 [M^+^] (calcd 234.2479). Analysis of the ^1^H NMR, ^13^C NMR and HMBC data of compound **2** revealed the characteristic of phenylpropanoid, including 1,4-disubstituted benzene ring (Table 3 and Fig. 4). The ^1^H NMR spectrum of compound **2** displayed two set of doublets δ_H_ 7.03 (2H, *d*, *J* = 8.6) and 7.38 (2H, *d*, *J* = 8.6) corresponding to two protons each, attributable to H-3/H-5 and H-2/H-6 respectively. Two methyl singlets at δ_H_ 2.29 (3H, *s*) and δ_H_ 2.10 (3H, *s*) corresponding to the methyl groups attached directly with the ester carbonyl groups, C-5′ and C-2″ respectively. In aromatic region, an AX spin system at δ_H_ 6.22 and 6.60 with a spin coupling of 15.9 Hz, which can easily be assigned to the protons H-2′ and H-1′ of the *trans* olefinic bond. The ^13^C NMR spectrum reveals a typical pattern of *para*-substituted aromatic compound. The long-range correlations between H-3′ and the carbonyl carbon at δ_C_ 170.7 (C-4′), H-1′ and C-3′ (δ_C_ 64.8), H-2 and C-1′, C-4 in HMBC spectrum (Fig. 4) confirmed the connection of a 1,4-disubstituted benzene ring with C-1′. The presence of two acetyl groups in these phenylpropanoid were deduced from the proton signals at δ_H_ 2.10 (3H, *s*) and δ_H_ 2.29 (3H, *s*) and the carbon signals at δ_C_ 169.3 (C-1″), 20.9 (C-2″), 170.7 (C-4′) and 21.0 (C-5′). The locations of the acetyl group at C-3′ was determined by the ^3^J (C-H) correlation between H-3′ and C-4′. Thorough analysis of the observed data and comparison with the literatures (Matsuda *et al.* 2003a,b) led to the deduction that compound **2** is *trans*-*p*-coumaryl diacetate.

Compound **3** was isolated as yellowish oil. The GC-EIMS of compound **3** revealed a molecular ion peak corresponding to [M^+^] at m/z 192 indicating the molecular formula C_11_H_12_O_3_. The IR spectrum showed absorption band at 3400 cm^−1^ (OH) and 1737 cm^−1^ (C=O ester). The ^1^H NMR and ^13^C NMR spectroscopic data of compound **3** (Table 3) indicated phenylpropanoid skeleton, which is similar to that of compound **2**; both have acetyl moiety at C-3′ (δ_C_ 64.8 in **2**; 63.6 in **3**) and *trans* double bond (δ_C_ 133.0, 121.6 in **2**; 130.1, 128.7 in **3**) directly attached to 1,4-disubstituted benzene ring. The principal difference in 1D NMR spectra of compound **3** was that with the replacement of acetyl moiety with hydroxy bearing quaternary carbon at C-4 (δ_C_ 134.5). The structure was further conformed by HMBC (Fig. 4) correlation between (H-5/C-4′; H-3′/C-2′; H-2′/C-3′, C-1′; H-1′/C-3′, C-2, C-6; H-3, H-5/C-4, C-1; H-2, H-6/C-3, C-5, C-1′, C-1) respectively. Similarly, the doublet attributed to H-1′ showed *J* = 15.9 Hz, which is an indicative of *trans* configuration at C-1′/C-2′. Thus, upon comparison of the obtained data and the literature values (Matsuda *et al.* 2003a,b; 2005), one may conclude that compound **3** is *p*-hydroxycinnamyl acetate.

Compound **4** was isolated as yellow oil. The GC-EIMS of compound **4** revealed a molecular ion peak corresponding to [M^+^] at m/z 192 indicating the molecular formula C_11_H_12_O_3_. The ^1^H NMR of compound **4** showed all features of chavicol acetate. The IR spectrum displayed absorption bands at 3080 cm^−1^ (OH), 1756 cm^−1^ (aliphatic ester C=O) and 1201 cm^−1^ (ester moiety C-O). The ^1^H NMR spectrum displayed the typical AA′XX′ pattern of a *para*-substituted benzene ring (Table 4). A strong singlet at δ_H_ 2.32 (3H, *s*) indicated a methyl group attached directly with the ester carbonyl group. Existence of a doublet at δ_H_ 5.18 (1H, *d*, *J* = 9.3) is belong to the methine group, H-1′ which directly attached to the benzene ring, the hydroxyl group and the ethylene group. Furthermore, the doublet at δ_H_ 5.32 might be attributable to ethylene proton H-3′a (1H, *d*, *J* = 17.1) while H-2′ emerged as a multiplet at 6.01 (1H, *m*). A set of doublets corresponding to four proton signals of H-2/H-6 and H-3/H-5 were appeared at δ_H_ 7.36 (2H, *d*, *J* = 8.6) and δ_H_ 7.05 (2H, *d*, *J* = 8.5) respectively. The ^13^C NMR spectrum of compound **4** showed the presence of 11 carbon atoms. The DEPT experiment indicated the presence of one ethyl, one CH_2_, six CH groups and three quaternary carbons. The ^13^C NMR spectrum of compound **4** also showed all the features of chavicol acetate (Table 4). The ^13^C NMR spectrum indicates the presence of ethylene carbon (δ_C_ 115.4 and 140.1), carbonyl group (δ_C_ 169.5) and methyl signal at δ_C_ 21.1. Further it also displayed signal at δ_C_ 140.0 and δ_C_ 150.1 due to quaternary carbon corresponding to *para*-disubstituted benzene ring. Meanwhile, the δ_C_ 140.1 is assignable to methine carbon C-2′. The complete assignment of protons and carbons was assisted by HMBC, COSY and HSQC experiments. The carbon skeletons suggested by several diagnostic correlations (H-2″/C-4, C-1″; H-1′/C-3′, C-2, C-6, C-2′; H-3′/C-1′, C-2′; H-2′/C-1′; H-3, H-5/C-1, C-4; H-2, H-6/C-1′, C-4) and ^1^H-^1^H-COSY (H-2/H-3; H-5/H-6; H-2′/H-3′). All the key HMBC correlations are depicted in Fig. 4. Based on these data and comparison with literature values (Jansen & Scheffer 1985), compound **4** was identified as (*S*)-4-(1-hydroxyally) phenyl acetate, a known phenylpropanoid and trivially named as 1′*S*-1′-hydroxychavicol acetate.

Compound **5** was obtained as yellowish oil. The IR spectrum exhibited absorption bands of carbonyl group at 1683 cm^−1^ (aldehyde) and hydroxyl group at 3369 cm^−1^. The molecular formula C_7_H_6_O_2_ was deduced on the basis of GC-EIMS (m/z 122 [M^+^]; calcd 122.1213). The ^1^H NMR data showed at δ_H_ 6.95 (2H, *d*, *J* = 8.8) and δ_H_ 7.81 (2H, *d*, *J* = 8.3) showed the presence of *para*-substituted benzene ring and methine signal at δ_H_ 9.87 (1H, *s*) (Table 4). The ^13^C NMR spectra revealed the presence of aldehyde conjugated with aromatic ring, resonating at δ_C_ 191.4 and a quaternary carbon linked with OH group, confirmed by the signal at δ_C_ 161.8. The remaining five carbon signal are for methine unit at 1,4-disubstituted benzene ring (δ_C_ 116.1 x 2 and 132.6 x 2) and a quaternary carbon at δ_C_ 129.7 (C-1) (Table 4). Comparison of ^1^H NMR and ^13^C NMR data of compound **5** with those of the known compound indicated that this compound was *p*-hydroxybenzaldehyde.

Compounds **6** and **7** were obtained as a mixture which is a white crystalline substance. These substances give the positive test to Salkowski and Liebermann-Burchard reagent for steroidal nucleus (Jain *et al.* 2009; Habib *et al.* 2007). The mass spectrum displays the typical pattern of polycyclic compound with many CH_2_ and CH groups. The spectrum showed a parent molecular ion [M^+^] peak at m/z 412 and 414, which corresponded to the molecular formula C_29_H_48_O and C_29_H_50_O respectively. The fragmentation peaks for compound **6** observed at m/z 412, 369, 327, 300, 271, 199, 173, 145 and 119, while m/z 414, 382, 273, 213, 187, 159, 133, 105, 81 and 55 for compound **7**. The IR spectroscopic analysis led to observation of some important bands 3430 cm^−1^ (hydroxyl stretching), 1653 cm^−1^ (C=C absorption), 1463 cm^−1^ (cyclic (CH_2_)_n_) and 1378 cm^−1^ (–CH_2_(CH_3_)_2γ_). The absorption frequency at 1049 cm^−1^ signifies cycloalkane. Compounds **6** and **7** have an identical sterol skeleton. Thus, the ^1^H and ^13^C NMR spectrums are similar to one another (Table 5). The ^1^H NMR spectrum of both compounds have revealed the existence of singlets at δ_H_ 0.67 and δ_H_ 1.00 corresponding to H-18/H-18′ and H-19/H-19′ methyl protons. Other methyl protons (H-21/H-21′, H-26/H-26′, H-27/H-27′ and H-29/H-29′) resonated between δ_H_ 0.65 to δ_H_ 1.00. The H-3/H-3′ protons appeared as a multiplet at δ_H_ 3.50, and H-6/H-6′ olefinic proton signals appeared as a multiplet at δ_H_ 5.33. The most significant difference on the ^1^H NMR chemical shift of these two molecules was the olefinic proton signals for H-22 and H-23 of compound **6**, which resonated as doublet of doublet signals at δ_H_ 5.17 (1H, *dd*, *J*_22_ = 8.7, *J*′_22_ = 15.6) and δ_H_ 5.03 (1H, *dd*, *J*_23_ = 8.7, *J*′_23_ = 15.6) respectively. The C-22′ and C-23′ ethylene proton signals of compound **7** resonated as multiplets in the region of δ_H_ 0.90 to δ_H_ 1.30. The rest of the protons resonated as multiplets in the region of δ_H_ 0.70 to δ_H_ 3.50. Many signals of both compounds were overlapping and gave the same ^1^H NMR signals. Since compounds **6** and **7** have an identical sterol skeleton, the ^13^C NMR spectrum for both compounds showed similar profile. The main different between compounds **6** and **7** were the signals of C-22/C-22′ and C-23/C-23′. For compound **6**, the *sp^2^* carbons; C-22 and C-23 resonated at δ_C_ 138.3 and δ_C_ 129.2 respectively. While for compound **7**, the two carbons resonated at δ_C_ 33.9 and δ_C_ 26.0 respectively. Another important signal was observed at 71.8 ppm corresponded to C-3 β-hydroxy group. The presence of the double bond also moved C-20, C-21, C-24, C-25 and C-28 further downfield at δ_C_ 40.5, 21.1, 51.2, 31.9 and 25.4 respectively as compared to that of compound **7**, which showed the signals at δ_C_ 36.1, 21.1, 45.8, 29.1 and 23.0 for C-20′, C-21′, C-24′, C-25′ and C-28′ respectively. From the above observations and comparison of the obtained spectral data with the literature values (Jayaprakasha *et al.* 2007; Forgo & Kövér 2004), compounds **6** and **7** were found to be stigmasterol and β-sitosterol, respectively.

**Figure 5:**
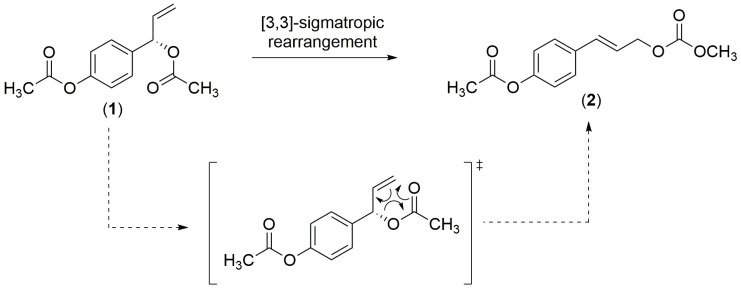
Chemical transformation of 1′*S*-1′-acetoxychavicol acetate (1).

A major compound, compound **1** was proceeded to the chemical transformation. Treatment of compound **1** with sodium acetate in acidic media afforded the rearrangement derivative, whose spectroscopic data were identical with those of compound **2**. Thus, the biosynthesis and the transformation of compound **2** was confirmed via [3,3]-sigmatropic rearrangement (Fig. 5). This transformation had been done because both compounds (i.e compounds **1** and **2**) were a pair of isomers, and isolated as a major component from this plant.

Three isolated compounds **1**, **2** and **5** were evaluated for their antimicrobial activity against MRSA using broth microdilution technique. The minimum inhibitory concentration (MIC) was used to evaluate the potential compounds as inhibitory agents against the strains of MRSA. Tetracycline was included as comparison for antimicrobial potential. Based on the results obtained in Table 6, the range of MIC values for all tested compounds is from 0.25 to 4.0 mg/mL. Compound **1** showed the good antimicrobial activity against the strain of MRSA with MIC value of 0.5 mg/mL. Meanwhile, compounds **2** and **5** exhibited moderate activity with MIC value between 1.0 and 2.0 mg/mL.

## CONCLUSION

The phytochemical investigation of the rhizomes of cultivate Alpinia conchigera Griff has led to the isolation of seven compounds namely 1′S-1′-acetoxychavicol acetate (**1**), *trans*-*p*-coumaryl diacetate (**2**), *p*-hydroxycinnamyl acetate (**3**), 1′*S*-1′-hydroxychavicol acetate (**4**), *p*-hydroxybenzaldehyde (**5**), stigmasterol (**6**) and β-sitosterol (**7**). Compounds **1**, **2** and **5** were screened for antimicrobial activity against MRSA ATCC 43300. Compound **1** exhibited good antimicrobial activity with MIC value of 0.5 mg/mL. It was also shown that compound **1** was a major compound from the rhizomes of *Alpinia conchigera* Griff. and can be further evaluated as a potential candidate for antimicrobial treatment against MRSA. These results provide some evidence to support the traditional application of this herb in treatment of skin infection as reported in Kelantan, East Coast Malaysia (Ibrahim *et al.* 2000).

## Figures and Tables

**Table 1:** Weight and percentage of crude extracts from the rhizomes of *Alpinia conchigera* Griff.

Plant material	Crude extract	Weight of crude extract (g)	Yield of crude extract (%)
Rhizomes	*n*-hexane	1.95	0.13
	Dichloromethane	9.60	0.64
	Methanol	11.7	0.78

**Table 2:** Chemical compounds isolated from the rhizomes of *Alpinia conchigera* Griff.

Crude extracts	Name of compound	% yield of compounds
*n*-hexane	1′*S*-1′-acetoxychavicol acetate (**1**)	25.64
	*trans*-*p*-coumaryl diacetate (**2**)	7.91
	*p*-hydroxycinnamyl acetate (**4**)	0.05
	*p*-hydroxybenzaldehyde (**5**)	0.07
Dichloromethane	1′*S*-1′-acetoxychavicol acetate (**1**)	23.11
	*trans*-*p*-coumaryl diacetate (**2**)	2.20
	1′*S*-1′-hydroxychavicol acetate (**4**)	0.01
	Stigmasterol (**6**)	5.21
	*β*-sitosterol (**7**)	

**Table 3:** ^1^H (500 MHz) and ^13^C (125 MHz) NMR data of compounds **1**–**3** (CDCl_3_).

Position	1		2		3	
		
	δ_H_ (*J* in Hz)	δ_C_	δ_H_ (*J* in Hz)	δ_C_	δ_H_ (*J* in Hz)	δ_C_
1	-	136.5	-	133.9	-	150.1
2	7.33 (2H, d, *J* = 8.8)	128.5	7.38 (2H, d, *J* = 8.6)	127.5	7.38 (2H, d, *J* = 8.8)	127.4
3	7.03 (2H, d, *J* = 8.8)	121.7	7.03 (2H, d, *J* = 8.6)	121.5	7.03 (2H, d, *J* = 8.5)	121.7
4	-	150.5	-	150.3	-	134.5
5	7.03 (2H, d, *J* = 8.8)	121.7	7.03 (2H, d, *J* = 8.6)	121.5	7.03 (2H, d, *J* = 8.5)	121.7
6	7.33 (2H, d, *J* = 8.8)	128.5	7.38 (2H, d, *J* = 8.6)	127.5	7.38 (2H, d, *J* = 8.8)	127.4
1′	6.23 (1H, d, *J* = 5.8)	75.6	6.60 (1H, d, *J* = 15.9)	133.0	6.58 (1H, d, *J* = 15.9)	130.1
2′	5.98 (1H, m)	136.1	6.22 (1H, m)	121.6	6.32 (1H, m)	128.7
3′	5.22 (2H, dd) (*J* = 10.5, *J* = 14.6)	117.2	4.70 (2H, d, *J* = 5.1)	64.8	4.31 (2H, d, *J* = 5.6)	63.6
4′	-	169.7	-	170.7	-	169.5
5′	2.08 (3H, s)	21.2	2.29 (3H, s)	21.0	2.30 (3H, s)	21.1
1″	-	169.4	-	169.3		
2″	2.27 (3H, s)	21.3	2.10 (3H, s)	20.9		

**Table 4:** ^1^H (500 MHz) and ^13^C (125 MHz) NMR data of compound **4**–**5** (CDCl_3_).

Position	4		5	
	
	δ_H_ (*J* in Hz)	δ_C_	δ_H_ (*J* in Hz)	δ_C_
1	-	140.0	-	129.7
2	7.36 (2H, d, *J* = 8.6)	127.3	6.97 (2H, d, *J* = 8.6)	116.1
3	7.05 (2H, d, *J* = 8.5)	121.5	7.81 (2H, d, *J* = 8.6)	132.6
4	-	150.2	-	161.8
5	7.05 (2H, d, *J* = 8.5)	121.5	7.82 (2H, d, *J* = 8.6)	132.6
6	7.36 (2H, d, *J* = 8.6)	127.6	6.97 (2H, d, *J* = 8.6)	116.1
1′	5.18 (1H, d, *J* = 9.3)	74.7	9.82 (1H, s)	191.4
2′	6.01 (1H, m)	140.2		
3′a	5.18 (1H, d, *J* = 9.3) 5.32 (1H, d, *J* = 17.1)	115.2		
1″	-	169.4		
2″	2.32 (3H, s)	21.2		

**Table 5:** ^1^H (500 MHz) and ^13^C (125 MHz) NMR data of compound **6**–**7** (CDCl_3_)

Position	6		7	
	
	δ_H_ (*J* in Hz)	δ_C_	δ_H_ (*J* in Hz)	δ_C_
1	1.82 (1H, m) 1.06 (1H, m)	37.2	1.82 (1H, m) 1.06 (1H, m)	37.2
2	1.82 (1H, m) 1.50 (1H, m)	31.6	1.82 (1H, m) 1.50 (1H, m)	31.6
3	3.50 (1H, m)	71.8	3.50 (1H, m)	71.8
4	2.29 (1H, m) 2.22 (1H, m)	42.3	2.29 (1H, m) 2.22 (1H, m)	40.2
5	-	140.7	-	140.7
6	5.33 (1H, m)	121.7	5.33 (1H, m)	121.7
7	1.98 (1H, m) 1.50 (1H, m)	31.9	1.98 (1H, m) 1.50 (1H, m)	31.9
8	1.46 (1H, m)	31.9	1.46 (1H, m)	31.9
9	0.92 (1H, m)	50.1	0.92 (1H, m)	50.1
10	-	36.5	-	36.5
11	1.50 (1H, m)	21.1	1.50 (1H, m)	21.1
12	1.98 (1H, m) 1.15 (1H, m)	39.7	1.98 (1H, m) 1.15 (1H, m)	39.8
13	-	42.3	-	42.3
14	1.01 (1H, m)	56.8	1.01 (1H, m)	56.7
15	1.56 (1H, m) 1.06 (1H, m)	24.3	1.56 (1H, m) 1.06 (1H, m)	24.3
16	1.65 (1H, m)	28.9	1.65 (1H, m)	28.2
17	1.13 (1H, m)	55.9	1.13 (1H, m)	56.0
18	0.67 (3H, s)	12.0	0.67 (3H, s)	11.8
19	1.00 (3H, s)	19.4	1.00 (3H, s)	19.4
20	2.01 (1H, m)	40.5	1.35 (1H, m)	36.1
21	1.01 (3H, s)	21.1	0.92 (3H, s)	21.1
22	5.17 (1H, dd) (*J*_22_ = 8.7, *J*′_22_ = 15.6)	138.3	1.01 (1H, m) 1.30 (1H, m)	33.9
23	5.03 (1H, dd) (*J*_23_ = 8.7, *J*′_23_ = 15.6)	129.2	1.15 (1H, m)	26.0
24	1.52 (1H, m)	51.2	0.92 (1H, m)	45.8
25	1.53 (1H, m)	31.9	1.65 (1H, m)	29.1
26	0.83 (3H, d, *J*_26_ = 6.5)	21.2	0.81 (3H, d, J_26_= 6.5)	19.8
27	0.78 (3H, d, *J*_27_ = 6.5)	19.0	0.83 (3H, d, *J*_27_ = 6.5)	18.8
28	1.15 (1H, m) 1.43 (1H, m)	25.4	1.24 (1H, m)	23.0
29	0.81 (3H, t, *J*_29_ = 7.8)	12.2	0.85 (3H, t, *J*_29_ = 7.8)	12.0

**Table 6:** Antimicrobial activity of isolate compounds against strain of MRSA ATC 43300.

Chemical compounds	MIC (mg/mL)
Tetracycline (control)	0.0005
1′S′-1′-acetoxychavicol acetate (**1)**	0.5
*trans-p*-coumaryl diacetate (**2**)	1.0
*p*-hydroxybenzaldehyde (**5**)	2.0

*Note*: MIC = minimum inhibitory concentration

## References

[b1-tlsr-31-1-159] Awang K, Azmi MN, Aun LII, Aziz AN, Ibrahim H, Nagoor NH (2010). The apoptotic effect of 1′*S*-1′-acetoxychavicol acetate from *Alpinia conchigera* on human cancer cells. Molecules.

[b2-tlsr-31-1-159] Aziz AN, Ibrahim H, Syamsir DR, Mohtar M, Vejayan M, Awang K (2013). Antimicrobial compounds from *Alpinia conchigera*. Journal of Etnopharmacology.

[b3-tlsr-31-1-159] Azuma H, Miyasaka K, Yokotani T, Tachibana T, Kojima-Yuasa A, Matsui-Yuasa I, Ogino K (2006). Lipase catalyzed preparation of optically active 1′-acetoxychavicol acetate and their structure activity relationships in apoptotic activity against human leukemia HL-60 cells. Bioorganic and Medicinal Chemistry.

[b4-tlsr-31-1-159] Baba J, Inabo HI, Umoh VJ, Olayinka AT (2016). Antibiotic resistance patterns of methicillin-resistance *Staphylococcus aureus* (MRSA) isolated from chronic skin ulcer of patients in Kaduna state, Nigeria. IOSR Journal of Pharmacy.

[b5-tlsr-31-1-159] Burkhill IH (1966). A dictionary of the economic products of the Malay Peninsular.

[b6-tlsr-31-1-159] Forgo P, Kövér KE (2004). Gradient enhanced selective experiments in the ^1^H NMR chemical shift assignment of the skeleton and side-chain resonances of stigmasterol, a phytosterol derivative. Steroids.

[b7-tlsr-31-1-159] Habib MR, Nikkon F, Rahman M, Haque ME, Karim MR (2007). Isolation of Stigmasterol and β-sitosterol from methanolic extract of root bark of *Calotropis gigantea* (Linn). Pakistan Journal of Biological Sciences.

[b8-tlsr-31-1-159] Hasima N, Aun LII, Azmi MN, Aziz AN, Thirthagiri E, Ibrahim H, Awang K (2010). 1′S-1′-acetoxyeugenol acetate: A new chemotherapeutics natural compound against MCF-7 human breast cancer cells. Phytomedicine.

[b9-tlsr-31-1-159] Henderson MR (1954). Malayan wild flowers: Monocotyledons.

[b10-tlsr-31-1-159] Ibrahim H, Chooi OH, Hassan R (2000). Ethnobotanical survey of the ginger family in selected Malay villages in Peninsular Malaysia. Malaysia Journal of Science.

[b11-tlsr-31-1-159] Ibrahim H, Awang K, Ali NAM, Malek SNA, Jantan I, Syamsir DR, Tohar N, Aziz AN (2008). Selected Malaysian aromatic plants and their essential oil component.

[b12-tlsr-31-1-159] Ibrahim H, Aziz AN, Syamsir DR, Ali NAM, Mohtar M, Ali RM, Awang K (2009). Essential oils of *Alpinia conchigera* Griff. and their antimicrobial activities. Food Chemistry.

[b13-tlsr-31-1-159] Jain PS, Bari SB, Surana SJ (2009). Isolation of Stigmasterol and Sitosterol from petroleum ether extract of woody stem of *Abelmoschus manihot*. Asian Journal of Biological Sciences.

[b14-tlsr-31-1-159] Janssen AM, Scheffer JC (1985). Acetoxychavicol acetate, an antifungal component of *Alpinia galanga*. Planta Medica.

[b15-tlsr-31-1-159] Jayaprakasha GK, Mandadi KK, Poulose SM, Jadegoud Y, Nagana Gowda GA, Patil BS (2007). Inhibition of colon cancer cell growth and antioxidant activity of bioactive compounds from *Poncirus trifoliata* (L.) Raf. Bioorganic Medicinal Chemistry.

[b16-tlsr-31-1-159] Lakhundi S, Zhang K (2018). Methicillin resistant *Staphylococcus aureus*: Molecular characterization, evolution, and epidemiology. Clinical Microbiology Review.

[b17-tlsr-31-1-159] Matsuda H, Pongpiriyadacha Y, Morikawa T, Ochi M, Yoshikawa M (2003a). Gastroprotective effects of phenylpropanoids from the rhizomes of *Alpinia galanga* in rats: structural requirements and mode of action. European Journal of Pharmacology.

[b18-tlsr-31-1-159] Matsuda H, Morikawa T, Managi H, Yoshikawa M (2003b). Antiallergic principles from *Alpinia galanga*: structural requirements of phenylpropanoids for inhibition of degranulation and release of TNF-α and IL-4 in RBL-2H3 cells. Bioorganic & Medicinal Chemistry Letters.

[b19-tlsr-31-1-159] Matsuda H, Ando S, Morikawa T, Kataoka S, Yoshikawa M (2005). Structure-activity relationships of 1′*S*-1′-acetoxychavicol acetate for inhibitory effect on NO production in lipopolysaccharide-activated mouse peritoneal macrophages. Bioorganic & Medicinal Chemistry Letters.

[b20-tlsr-31-1-159] Ridley HN (1909). Material for a flora of the Malayan Peninsular.

[b21-tlsr-31-1-159] Smith RM (1990). Alpinia (Zingiberaceae): A proposed new infragenesis classification. Edinburgh Journal of Botany.

[b22-tlsr-31-1-159] Victorio CP (2011). Therapeutic value of the genus *Alpinia*, Zingiberaceae. Brazilian Journal Pharmacology.

[b23-tlsr-31-1-159] Watanabe N, Kataoka T, Tajika T, Uramoto M, Magae J, Nagai K (1995). 1′-acetoxychavicol acetate as an inhibitor of phagocytosis of macrophages. Bioscience Biotechnology and Biochemistry.

[b24-tlsr-31-1-159] Wongsatit C, Ampol B (2003). Ethnomedical uses of Thai Zingiberaceous plant. Annual Journal Herbal.

[b25-tlsr-31-1-159] Yasuhara T, Manse Y, Morimoto T, Qilong W, Matsuda H, Yoshikawa M, Muraoka O (2009). Acetoxybenzhydrols as highly active and stable analogues of 1′*S*-1′-acetoxychavicol, a potent antiallergic principal from *Alpinia galangal*. Bioorganic and Medicinal Chemistry Letters.

